# The Role of Line-Field Confocal Optical Coherence Tomography in Detecting Extramammary Paget Disease Recurrences: A Pilot Diagnostic Study

**DOI:** 10.3390/diagnostics14141562

**Published:** 2024-07-19

**Authors:** Gwendoline Diet, Clément Lenoir, Margot Fontaine, Lucas Boussingault, Carmen Orte Cano, Lyna Mtimet, Danielle Liénard, Dilara Sanak, Florine Moulart, Dana Bernardi, Anne-Laure Trepant, Javiera Perez-Anker, Susana Puig, Josep Malvehy, Elisa Cinotti, Linda Tognetti, Pietro Rubegni, Jean-Luc Perrot, Véronique Del Marmol, Mariano Suppa

**Affiliations:** 1 Department of Dermatology, Hôpital Erasme, Université Libre de Bruxelles (ULB), HUB, 1070 Brussels, Belgium; 2 Melanoma Unit, Hospital Clinic Barcelona, University of Barcelona, 08036 Barcelona, Spain; 3 Department of Dermatology, Institut Jules Bordet, Université Libre de Bruxelles (ULB), HUB, 1070 Brussels, Belgium; 4 Department of Pathology, Erasme University Hospital, Université Libre de Bruxelles (ULB), 1070 Brussels, Belgium; 5 Centro de Investigación Biomédica en Red de Enfermedades Raras (CIBERER), Carlos III Health Institute, 28029 Barcelona, Spain; 6 Unit of Dermatology, Department of Medical, Surgical and Neurological Sciences, University of Siena, 53100 Siena, Italy; 7 Groupe d’Imagerie Cutanée Non Invasive (GICNI) of the Société Française de Dermatologie (SFD), 42055 Paris, France; 8 Department of Dermatology, University Hospital of Saint-Etienne, 42055 Saint-Etienne, France

**Keywords:** extramammary Paget disease, non-invasive imaging techniques, line-field confocal optical coherence tomography, LC-OCT, recurrence, diagnostic accuracy

## Abstract

Extramammary Paget disease (EMPD) is an uncommon adenocarcinoma of apocrine gland-rich areas, presenting significant diagnostic challenges due to its nonspecific clinical appearance and frequent misidentification as benign, inflammatory skin conditions. Traditional diagnostic methods such as biopsy are invasive and uncomfortable, often required repeatedly due to high recurrence rates. Dermoscopy and non-invasive imaging techniques have been used but provide limited diagnostic accuracy due to their constraints in depth penetration and resolution. Recent advancements in imaging technologies, such as line-field confocal optical coherence tomography (LC-OCT), show promise in enhancing diagnostic precision while minimizing invasive procedures. LC-OCT merges high-resolution imaging with deep penetration capabilities, capturing detailed horizontal and vertical skin images akin to histopathology. This study evaluated the diagnostic performance of LC-OCT in detecting EMPD and its recurrence in 17 clinically suspicious anogenital regions, belonging to six patients. Data were collected prospectively at the patient’s bedside by an LC-OCT expert with poor training for EMPD, and, then, reviewed retrospectively by an independent LC-OCT expert with adequate training for EMPD and no concerns about time. The prospective examination yielded 64.7% accuracy (11 true results out of 17 total cases), 71.4% sensitivity (10 true positives out of 14 actual positives), and 33.3% specificity (1 true negative out of 3 actual negatives). The retrospective analysis achieved 94.1% accuracy (16 true results out of 17 total cases), 100% sensitivity (14 true positives out of 14 actual positives), and 66.7% specificity (2 true positives out of 3 actual positives), with the only false positive case being a difficult-to-diagnose concomitant presentation of a lichen sclerosus et atrophicus. Despite the need for specialized training, our results suggest that LC-OCT represents a valuable tool for accurately identifying EMPD and improving its management by reducing unnecessary biopsies. Further studies are needed to standardize its clinical application.

## 1. Introduction

Extramammary Paget disease (EMPD) is a relatively uncommon form of adenocarcinoma localized on apocrine gland-rich areas and first described by Henry Radcliffe Crocker in 1889 [1]. It typically occurs in individuals in their sixth decade of life and affects women more frequently than men worldwide, with an Asian exception where occurrence is equal across sexes [2]. It appears clinically as slow-growing, demarcated, thickened, erythematous plaques, less commonly ulcerated, eroded, or crusted due to recurrent trauma from pruritus or secondary infection, especially in long-standing disease [2,3]. EMPD often poses significant diagnostic challenges due firstly to its nonspecific clinical presentation [2,3], frequently resembling benign, inflammatory, or infectious skin conditions and secondly to its frequent ill-defined [3] presentation affecting delicate areas such as genitalia [2,3,4,5]. This often leads to delayed diagnoses or suboptimal treatment [2,3]. Furthermore, EMPD predominantly affects the perianal and genital regions and rarely can also be ectopic [2,4,5], making its accurate diagnosis and monitoring not only crucial for effective management but also sensitive. Current diagnostic methods, primarily histopathology requiring biopsy [2,4,5], are invasive and uncomfortable for patients, especially in the context of a chronic pathology with a high rate of recurrence where multiple biopsies will be required throughout the course of the disease [2,3]. The morbidity and related costs associated with biopsies highlight the need for advancements in non-invasive, accurate diagnostic tools that can alleviate patient distress by minimizing invasive procedures and improving diagnostic timelines.

Among them, dermoscopy can only be considered as an adjunct tool for the diagnosis of EMPD due to the lack of microscopic information and the overlap of several dermoscopic criteria between EMPD and its mimickers [6,7].

Reflectance confocal microscopy (RCM) exclusively provides horizontal (*en face*) images of the skin, with ~200 μm penetration depth and 1 μm lateral resolution allowing for the perfect visualization of the cells [8]. Previous studies showed a strong correlation between RCM and histological findings confirming its ability to diagnose and differentiate EMPD from inflammatory or tumoral skin diseases [9,10]. A recent prospective study performed on 5 patients reported a 75% sensitivity and a 100% specificity, suggesting that this technique may potentially improve EMPD detection [11]. Nevertheless, the limited depth of examination and the need for specialized skills to interpret horizontal views restrict the deployment of this technology to only a limited number of specialized centers [8,12].

Conventional optical coherence tomography (c-OCT), offering both *en face* and cross-sectional imaging, provides a greater penetration depth (varying from 1 to 2 mm) and a lower axial and lateral resolution (around 15 μm) [13], which limits its utility in EMPD [14,15]. Conversely, full-field OCT (FF-OCT) achieves superior axial and lateral resolutions of 1.35 μm and 1 μm, respectively, though at a reduced penetration depth of 400 μm [16]. This enhanced resolution makes FF-OCT particularly suitable for capturing detailed images of epidermal layers, essential in the accurate diagnosis of dermatological conditions like EMPD. This advancement enables the description of EMPD patterns well correlated to histopathology [16]. However, given the limited number of publications and the absence of large clinical trials, only a few selected centers appear to be using this technology at present.

Although ultrasonographic characteristics of EMPD are not yet reliable for diagnostic purposes, high-frequency ultrasound (HF-US) represents an additional tool for the management of EMPD [17], helping to determine the extent of disease pre- and post-treatment [17,18].

Line-field confocal OCT (LC-OCT) is one of the latest non-invasive imaging technologies available on the market and offers a “virtual histology” of skin lesions [19]. This technology captures optical images in real time, both horizontally and vertically, with confocal-like resolution (axial resolution of 1.1 µm and lateral resolution of 1.3 µm) and conventional OCT-like depth of penetration (500 µm) [19]. Moreover, an integrated dermoscopic camera helps in navigating precisely into the lesion and in outlining margins. Merging the high-resolution benefits of RCM with the vertical imaging and deep penetration capabilities of OCT, LC-OCT has been the subject of numerous publications that explore a variety of dermatological disorders [20,21], emphasizing its value in the diagnosis of many skin conditions thus reducing the need for unnecessary biopsies. It has been reported that LC-OCT has greater diagnostic accuracy for skin carcinomas than clinical/dermoscopic evaluation both in studies [22] and in real-life settings [23,24]. However, to date, there are only two publications on EMPD imaged by LC-OCT, which describe features that closely resemble histopathological observations [25,26]. Di Stefani et al. [25] reported a single case of EMPD in the pubic area imaged with LC-OCT and described for the first time the LC-OCT correlates of the disease, namely nests and solitary cells, larger than normal keratinocytes, observed in epidermal layers and at the dermal–epidermal junction (DEJ). Delpuech et al. [26] reported a case of EMPD in the anal area and a case of mammary Paget disease and confirmed the previously described LC-OCT features of Paget disease.

Therefore, there is a current lack of data about the diagnostic performance of LC-OCT in detecting EMPD. This represents a significant gap in the literature, as LC-OCT seems to be a valuable tool for improving the clinical diagnostic accuracy of EMPD and the assessment of EMPD recurrence while reducing the number of avoidable biopsies, thanks to its resemblance to histopathological images, ease of use, and ability to perform live, localized dermoscopic imaging.

The primary objective of this study was to evaluate the diagnostic performance of LC-OCT in detecting EMPD and, specifically, its recurrence following surgical or medical treatment. For this purpose, we employed data collected from a patient’s bedside prospective setting complemented by a subsequent, retrospective, expert image revision.

## 2. Materials and Methods

We retrospectively included a cohort of patients with histopathologically proven EMPD referred to the Department of Dermatology of the Hôpital Erasme, Université Libre de Bruxelles (Brussels, Belgium) to rule out disease persistence or recurrence after diverse treatment approaches, including topical therapies (imiquimod) and surgical procedures. The patient evaluations spanned from February 2020 to January 2024. During this period, LC-OCT (DAMAE Medical, Paris, France) was used in each patient to evaluate clinically suspicious areas for the presence of EMPD prior to skin biopsy for confirmation or exclusion of EMPD diagnosis.

The study was structured into three distinct phases: (i) LC-OCT data were first prospectively collected at the patient’s bedside by an LC-OCT expert and methodically documented in the patient’s medical record; (ii) the imaged sites were subsequently biopsied for histopathological confirmation (deemed gold standard diagnostic test); and, finally, (iii) a subsequent retrospective evaluation of the previously collected LC-OCT images/videos was conducted by an independent LC-OCT expert, blinded to the histopathological data, who had a specific training based on two recent publications on LC-OCT for EMPD [25,26], which were not available during the initial prospective evaluation. This phase aimed to assess the diagnostic reliability and accuracy of LC-OCT in a blinded and controlled setting. Both LC-OCT experts (prospective and retrospective evaluation) had a 5-year experience with the technology and were aware of (unblinded to) the study topic because the study included only patients with histopathologically proven EMPD to rule out post-treatment disease persistence/recurrence.

In our assessment criteria, a site was considered to be affected by EMPD if Paget’s cells (PCs) were identified within the epidermis and at the DEJ during LC-OCT evaluation, as previously shown [25,26]. PCs were characterized by their distinct appearance as dark to black cells, noticeably larger than the adjacent keratinocytes in the epidermis. PCs can be found either singly or clustered, forming dark glandular structures that are mainly located at the level of the dermal–epidermal junction. The identification of these specific characteristics was crucial for confirming or disagreeing with the presence of disease at the evaluated sites. Conversely, a site was considered to be non-affected by EMPD (i.e., negative) if PCs were not identified during LC-OCT evaluation.

Categorical variables are presented with numbers with percentages, and continuous variables are presented with a median and range. Key statistical measures were calculated to evaluate the diagnostic performance of the study: sensitivity was determined as the proportion of true positives (TPs) out of the total actual positives (TPs + false negatives [FNs]); specificity as the proportion of true negatives (TNs) out of the total actual negatives (TNs + false positives [FPs]); positive predictive value (PPV) as the ratio of TPs to the total predicted positives (TPs + FPs); negative predictive value (NPV) as the ratio of TNs to the total predicted negatives (TNs + FNs); and accuracy as the proportion of all true results (both TPs and TNs) out of the total cases. Statistical analyses were performed using Microsoft^®^ Excel version 15.32 © 2017 (Microsoft Corporation, Redmond, Washington, DC, USA).

## 3. Results

Overall, we included 17 clinically suspicious anogenital regions indicative of EMPD, belonging to six patients (three females and three males) with a median age of 63 (38–72) years. Of these, 14 regions (82.4%) were histopathologically positive for either EMPD persistence or recurrence after the study biopsy, while 3 regions (17.6%) did not show histopathological signs of the disease.

**Figure 1:**
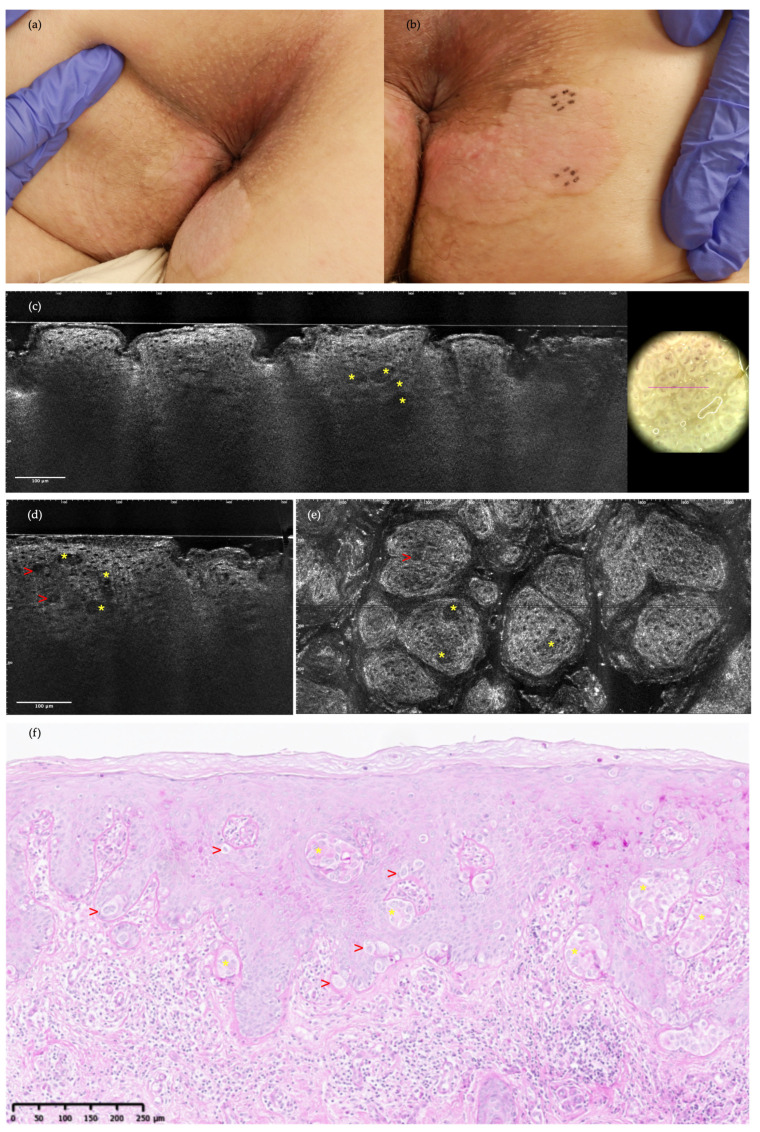
Clinical images, vertical and horizontal line-field confocal optical coherence tomography (LC-OCT), and the histopathology of extramammary Paget disease (EMPD) in a 38-year-old woman correctly identified both in the prospective and retrospective evaluations: (**a**) pinkish plaque of the peri-anal area previously treated with imiquimod; (**b**) circular markers indicate sites of scouting biopsies; (**c**,**d**) vertical and (**e**) horizontal LC-OCT frames reveal solitary Paget’s cells (PCs) characterized by a dark cytoplasm and a slightly bright nucleus (red arrows) and larger than neighboring keratinocytes, as well as glandular structures representing clusters of PCs (yellow asterisks); (**f**) histopathology (periodic acid Schiff) shows PCs across the epidermis, structured as single (red arrows) or nested cells (yellow asterisks).

During the initial prospective bedside evaluation (Table 1), 10 out of 14 positive cases were correctly identified, and 1 out of 3 negative cases was accurately recognized, resulting in 4 false negatives and 2 false positives. This phase concluded with an overall accuracy of 64.7%, a sensitivity of 71.4%, a specificity of 33.3%, a positive predictive value (PPV) of 83.3%, and a negative predictive value (NPV) of 20% (Figure 1).

**Figure 2:**
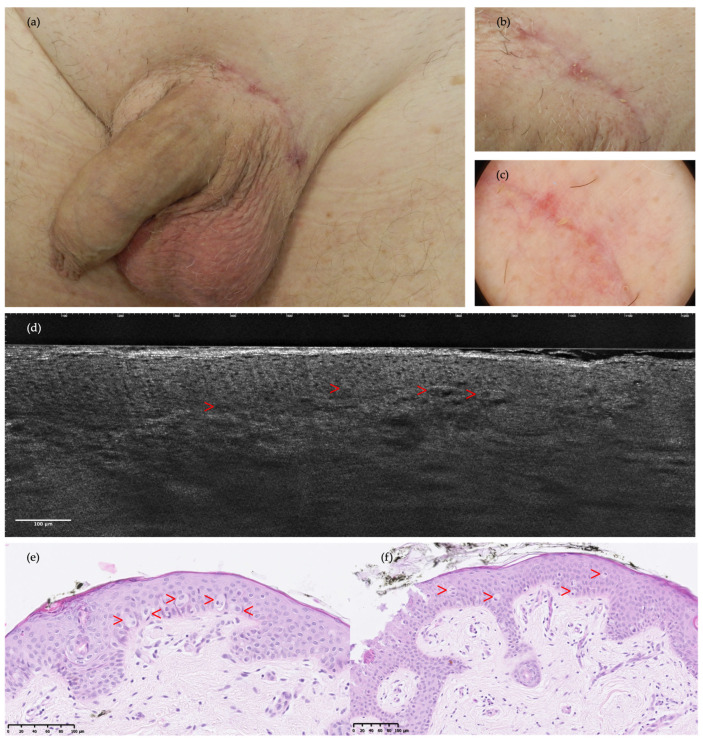
Clinical images, LC-OCT, and the histopathology of pubic EMPD in a 71-year-old man: (**a**,**b**) recurrent erythematous patches in a surgical scar with unclear margins from recent surgery; (**c**) dermoscopy presenting red-pink structureless areas and polymorphous vessels; (**d**) prospective LC-OCT examination was interpreted as negative, although, after reevaluation (retrospective settings), few solitary PCs were identified (red arrows) as prominent dark structures containing either bright centers or dark holes related to the nucleus; (**e**,**f**) histopathology specimens (hematoxylin and eosin) match with LC-OCT frames, showing PCs exclusively organized as pale isolated cells predominantly present at the dermal–epidermal junction level (red arrows).

The subsequent retrospective evaluation (Table 2) correctly identified all (14/14) positive cases and 2/3 negative cases (i.e., no false negative and 1 false positive case, also detected as such in the prospective evaluation setting). All four false negative cases that were detected by the first prospective evaluation were lesions that had undergone multiple surgical treatments but were then correctly identified as positive cases by the second retrospective evaluation (Figure 2). This yielded an improved diagnostic accuracy of 94.1%, with 100% sensitivity, 66.7 specificity, 93.3% PPV, and 100% NPV.

**Figure 3:**
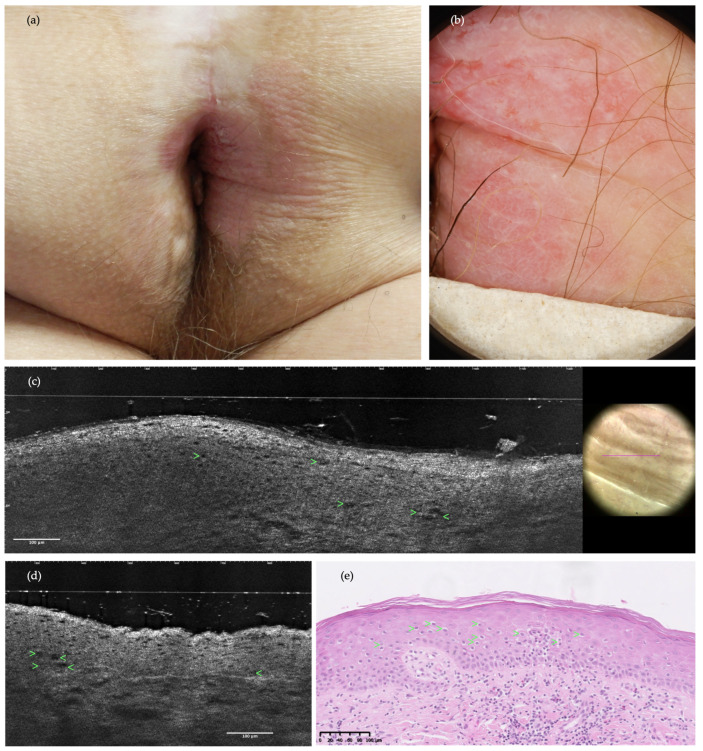
Clinical image, dermoscopy, LC-OCT, and the histopathology of the perianal area in a 72-year-old man with concomitant EMPD and lichen sclerosus atrophicus (LSA): (**a**) poorly delineated, atrophic, white, and erythematous plaque; (**b**) dermoscopy exhibiting milky-red structureless areas, and dotted and polymorphic vessels; (**c**,**d**) LC-OCT examination in both prospective and retrospective settings was misinterpreted as positive for EMPD recurrence, while histopathology pointed towards the diagnosis of LSA instead. The misinterpretation of EMPD was due to the visualization of vacuolized and enlarged keratinocytes (green arrows) looking similar to solitary PCs as they presented similar, but smaller central dark holes; (**e**) the H&E stained histopathological section shows vacuolized and enlarged keratinocytes (green arrows), which are indeed distinct from PCs as they are smaller in size.

The histopathological examination of the only remaining false positive case revealed the presence of lichen sclerosus et atrophicus (LSA)—a known comorbidity in this patient (a 72-year-old man with concomitant EMPD and LSA)—which was identified by the pathologist as the presence of a lymphocytic infiltrate and vacuolized keratinocytes within an edematous epidermis (spongiosis), which—albeit their smaller size—was mistaken for Paget’s cell infiltration in both LC-OCT assessment settings (Figure 3).

## 4. Discussion

The study at hand delves into the diagnostic challenges of EMPD, a condition known for its elusive presentation, and the critical need for timely and accurate detection to avoid disease progression [27]. As EMPD often mimics other dermatological conditions in its initial stages, an early differential diagnosis is crucial to ensure appropriate treatment, prevent disease progression, and avoid greater morbidity due to extensive surgery [28].

To date, there is considerable variability in non-invasive diagnostic techniques applied to EMPD [14] as well as a lack of standardized interpretation criteria. The findings from our study with LC-OCT offer promising insights into how this non-invasive technique can improve accuracy and reduce the need for biopsies.

In this preliminary diagnostic study, we assessed the accuracy of LC-OCT for detecting persistence (i.e., continued existence of the disease after diagnosis) or recurrence (i.e., relapse after a period of absence or remission) of EMPD in a cohort of patients with histopathologically proven EMPD. Two separate phases of data collection were conducted, initially by an LC-OCT expert with limited training in EMPD detection, who evaluated images and videos at the patient’s bedside (prospective setting), and then by an independent LC-OCT expert with sufficient training in EMPD detection, who evaluated the same material and had the time to do so properly (retrospective setting).

The retrospective evaluation, performed by definition under ideal and optimal conditions, produced a remarkable diagnostic accuracy of 94.1%, with 100% sensitivity and 66.7% specificity. Of note, the suboptimal specificity might be explained by the limited amount of true negative cases (only 3 out of 17) and by the fact that the only false positive was detected in a site concomitantly affected by LSA, in which one of the histopathological hallmarks of the disease—the inflammatory infiltrate—was mistaken for Paget’s cells on LC-OCT. To date, only two cases of clinically diagnosed LSA with biopsy-proven EMPD have been reported in the literature [29,30], and no case of concomitant diseases proven by biopsy has been described yet, which highlights the exceptional and consequently unusual nature of our case, which undoubtedly lead us to misinterpretation.

Non-invasive imaging techniques have been previously employed to differentiate EMPD from LSA. Dermoscopy of EMPD (milky-red areas and polymorphous vessels) and LSA [inverse follicular plugs and reduced vessel density (so-called “desertification”), not present in our case] were directly compared in one study [6]. Under RCM, LSA is characterized by the presence of dermal fibrosis corresponding to thick fiber-like structures together with a typical honeycomb pattern, dilated and irregular dermal papillae, and bright cells in the dermis [31]. Under c-OCT, LSA features sclerotic changes defined by a disorganized extracellular matrix, increased blood flow, and epidermal thinning [32]. Under HF-US, LSA is visualized as a hypoechoic band in the dermis [33]. None of the above-mentioned features were present in our case, neither during the dermoscopy nor LC-OCT, thus confirming this particularly difficult presentation. Although there are many illustrations and descriptions of non-invasive imaging modalities for EMPD and LSA, there is still insufficient data from large studies demonstrating their ability to clearly differentiate EMPD from LSA, and nor is there any data describing LSA in LC-OCT. To date, the only documented cases of LC-OCT related to LSA concern five cases of lichen planus (both belonging to interface dermatitis), which feature thickened stratum corneum and epidermis, hypergranulosis, and inflammatory bright cells at the level of the dermal–epidermal junction [34]. An explanation for the misdiagnosis of LSA as EMPD in LC-OCT could reside in two particular histopathological features of LSA [35]. These features involved in our case are a slight vacuolar alteration of keratinocytes and a focal lymphocyte exocytosis with spongiosis of the epidermis [35]: although smaller in diameter, these features resemble the dark epidermal structures corresponding to single PCs under LC-OCT examination, which prompted both the prospective and the retrospective observer to misdiagnose this very difficult case. 

In a previous diagnostic study with RCM [19] carried out on a population with a more balanced distribution (22 sites, of which there are 12 true positives and 10 true negatives), the authors found a 100% specificity, but none of their negative cases had concomitant inflammatory diseases and were, therefore, putatively less likely to be misinterpreted as false positive cases. Conversely, they only found a 75% sensitivity, as three false negative cases were identified on the lesion margins, deemed difficult locations as they were close to recent biopsies and due to the hypothetical lower density of PCs. This comparison highlights the unique strengths and limitations of different imaging techniques, underscoring the importance of selecting the appropriate method based on the specific clinical scenario and patient needs. A possible explanation of the different sensitivities (i.e., ability to find the disease) between RCM in the study of Yélamos et al. [11] and LC-OCT in our pilot study might reside in the ability of LC-OCT to ensure the comprehensive coverage of the examined area facilitated by its integrated dermoscopic camera and enabling us to precisely select a doubtful location for histopathological examination. This specific ability has already demonstrated remarkable efficacy in the field of oncology for basal cell carcinoma subtyping, as previously reported by Cappilli et al. [36].

Interestingly, our results confirm the need for good conditions for image interpretation as well as for a learning curve for the use of LC-OCT. Indeed, all diagnostic parameters improved from the preliminary prospective evaluation, performed by an operator considered poorly trained at that time and during the rush of a conventional consultation, to the more mature retrospective assessment, performed under ideal conditions. The improvement in diagnostic accuracy between the first and second imaging experts suggests a training effect. However, the second expert’s training focused solely on EMPD publications, potentially introducing bias by narrowing their diagnostic considerations. This could have led to a singular focus on EMPD without considering other differential diagnoses, which could be regarded as a limitation of the study. Of note, all four false negative cases of the first prospective evaluation (then correctly identified as positive cases by the second retrospective evaluation) had previously received multiple surgeries and were, therefore, more difficult to interpret during the consultation due to the conspicuous presence of scar tissue mirroring, which was what was found challenging in Yélamos et al.’s study [11]. The challenges associated with interpreting post-surgical changes emphasize the need for highly skilled operators who can discern subtle differences between disease recurrence and post-treatment changes. Possibly, the better conditions of the retrospective evaluation (better training and more time) allowed for the correct interpretation of these difficult cases but also represent the main limitation of this study, as all diagnoses were performed by a single skin imaging expert without any concern about the time employed to do so. Finally, the fact that different regions within the same patient were considered independent (i.e., a negative area in a patient with positive areas was regarded as truly negative) might hypothetically represent another limitation of the study. However, we believe this is not the case here, as the same assumption is considered valid in histopathology (i.e., the absence of Paget’s cells corresponds to the absence of EMPD).

The evident post-training improvement in diagnostic precision reinforces the role of LC-OCT as a powerful tool in the dermatological arsenal. The adoption of advanced imaging technologies such as LC-OCT in clinical settings not only enhances diagnostic accuracy but also appreciably improves the patient experience by reducing the need for invasive procedures. Indeed, the 100% sensitivity found in this study underscores the ability of LC-OCT to have at the same time good cellular resolution (thanks to the high numeric aperture of its microscope objective) and good lesion coverage (thanks to its coupling with an integrated dermoscopic camera), which potentially renders it a new, important candidate for the diagnosis and management of EMPD. Additionally, the integration of LC-OCT into clinical practice could potentially lead to an earlier detection and treatment of EMPD, which is critical for improving patient outcomes. Also, our data suggest that thorough training is imperative for the effective use of LC-OCT for EMPD, and it may be beneficial for future studies to establish standardized practice and examination protocols to maximize diagnostic accuracy for EMPD during real-life consultation settings. In particular, the possibility of underlying additional diseases or sampling bias needs a cautious approach in interpreting LC-OCT findings. Proper training and standardized protocols are essential to ensure that LC-OCT is used effectively and consistently across different clinical settings. Future larger studies including more numerous and better-distributed populations are needed to confirm these considerations.

In conclusion, this study represents a significant addition to the current body of evidence about non-invasive skin imaging for EMPD as it produced for the first time important data about the diagnostic performance of LC-OCT in detecting disease recurrence/persistence and highlighted the importance of correct training and working conditions for the LC-OCT evaluator.

## Figures and Tables

**Table 1:** Initial prospective bedside evaluation: True positives (TPs): 10 cases where both LC-OCT and histopathological evaluations assessed Paget’s cells (PCs) as positive. False negatives (FNs): 4 cases where LC-OCT was negative but histopathological evaluation was positive. False positives (FPs): 2 cases where LC-OCT was positive but histopathological evaluation was negative. True negatives (TNs): 1 case where both LC-OCT and histopathological evaluations were negative.

	Prospective LC-OCT Evaluation		
Histopathological Evaluation	Presence of PCs Assessed by the Expert	Absence of PCs Assessed by the Expert	
Presence of the disease	10 (TP)	4 (FN)	14
Absence of the disease	2 (FP)	1 (TN)	3
Total	12	2	17

**Table 2:** Retrospective LC-OCT evaluation: True positives (TPs): 14 cases where both LC-OCT and histopathological evaluation showed the presence of PCs. False negative (FN): 0 cases where LC-OCT failed to show PCs while histopathological evaluation showed disease presence. False positive (FP): 1 case where LC-OCT showed the presence of PCs, but histopathological evaluation showed the absence of disease. True negatives (TNs): 2 cases where LC-OCT showed the absence of PCs confirmed by histopathological evaluation.

	Retrospective LC-OCT Evaluation		
Histopathological Evaluation	Presence of PCs Assessed by the Expert	Absence of PCs Assessed by the Expert	
Presence of the disease	14 (TP)	0 (FN)	14
Absence of the disease	1 (FP)	2 (TN)	3
Total	15	2	17

## Data Availability

The data presented in this study are available upon request from the corresponding author due to privacy, legal, and ethical reasons.
